# Abrupt *p-n* junction using ionic gating at zero-bias in bilayer graphene

**DOI:** 10.1038/s41598-017-03264-0

**Published:** 2017-06-13

**Authors:** Sameer Grover, Anupama Joshi, Ashwin Tulapurkar, Mandar M. Deshmukh

**Affiliations:** 1 Department of Condensed Matter Physics and Materials Science, Tata Institute of Fundamental Research, Homi Bhabha Road, Mumbai, 400005 India; 2 Department of Electrical Engineering, Indian Institute of Technology Bombay, Mumbai, 400076 India

## Abstract

Graphene is a promising candidate for optoelectronic applications. In this report, a double gated bilayer graphene FET has been made using a combination of electrostatic and electrolytic gating in order to form an abrupt *p-n* junction. The presence of two Dirac peaks in the gating curve of the fabricated device confirms the formation of a *p-n* junction. At low temperatures, when the electrolyte is frozen intentionally, the photovoltage exhibits a six-fold pattern indicative of the hot electron induced photothermoelectric effect that has also been seen in graphene *p-n* junctions made using metallic gates. We have observed that the photovoltage increases with decreasing temperature indicating a dominant role of supercollision scattering. Our technique can also be extended to other 2D materials and to finer features that will lead to *p-n* junctions which span a large area, like a superlattice, that can generate a larger photoresponse. Our work creating abrupt *p-n* junctions is distinct from previous works that use a source–drain bias voltage with a single ionic gate creating a spatially graded *p-n* junction.

## Introduction

Graphene^1^ has unique optical^2–4^ and electronic^5^ properties which has made it a promising material for optoelectronic devices such as photodetectors. The creation of *p-n* junctions with tunable chemical potentials allows the transduction of light into electrical signals. In conventional electrostatic gating, a metallic gate separated by a dielectric is used. The maximum carrier density, typically 5 × 10^12^ cm^−2^ for silicon dioxide, is limited by the breakdown strength of the dielectric.

Electrolytic gating^6^ is an alternate technique that has the advantage of large achievable carrier densities, ~4 × 10^13^ cm^−2^. This is limited by the leakage current through the electrolyte^7^. The large capacitance is a result of the formation of an interfacial electrical double layer^8^ with a thickness of ~1 nm. Electrolytic gating has been used for tuning the carrier density in various semiconductors such as organic polymers^9^, carbon nanotubes^10^, and superconductors^11, 12^. Other non-tunable techniques for large doping density include chemical doping^13, 14^, exposure to electron beams^15^ or light^16, 17^, incorporating dopant atoms in the lattice^18, 19^ and other growth techniques^20^. Graphene can be electrolytically gated using different types of electrolytes such as aqueous solutions of salts^21^, solid polymer electrolytes of lithium or potassium salts in a polymer matrix^6^, ionic liquids^22^ and their gels^23^. Electrolytic gating has been used previously in order to explore properties such as superconductivity in MoS_2_^24^. The achievement of large carrier density is important for the study of properties of graphene below the Bloch-Grüneisen temperature^25^. The added advantage of this technique is that the ionic liquids have larger optical transmission compared to a metallic top gate.

**Figure 1:**
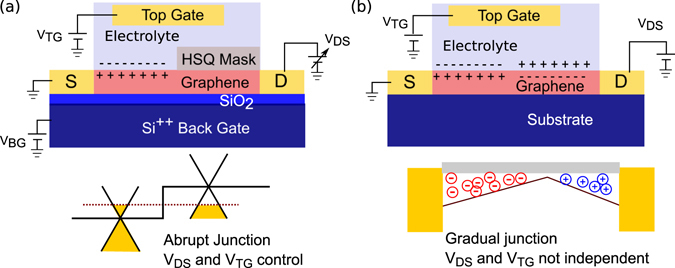
Comparison of the gating scheme discussed in this work with that used previously. **(a)** Schematic of our gating scheme and the corresponding band diagram with abrupt *p-n* junction and independent control of *V*_*DS*_. **(b)** Electrolytic gating scheme used in previous experiments^27, 28^ where a combination of *V*_*DS*_ and *V*_*GS*_ is used to create a junction which is not abrupt and does not allow independent control of *V*_*DS*_ and the barrier height.

In this article, we demonstrate the formation of a *p-n* junction in graphene using a combination of electrostatic and electrolytic gating at zero bias. Part of the graphene is covered with a protective layer of hydrogen silsesquioxane (HSQ) resist which prevents the electrolytic top gate from influencing the entire graphene region. Creating *p-n* junctions at zero bias using electrolyte gates is scalable and has not been done before. Graphene *p-n* junctions have previously been created using metallic gates and with electrolytic gating by putting ionic liquid drop on millimetre sized CVD graphene^26^ and by applying a drain-source bias comparable with the gate voltage^27^. Using the existing non-zero bias technique, an abrupt *p-n* junction is difficult to realize. With our technique, we can create an abrupt profile which is important for optoelectronic applications. Further, we have independent control of the source drain bias voltage and *p-n* junction barrier height and using a zero bias is possible. Table 1 summarizes work related to the formation of *p-n* junction by various techniques and establishes novel aspect of our work. Figure 1 shows a comparison of the abrupt *p-n* junction profile that we have formed and compared it to previous reports that use a source–drain bias voltage to create a gradual junction using electrolytic gating.

**Table 1:** Comparison of reports of formation of *p-n* junction in graphene and related 2-D materials.

Reference	Material Used	Study
Zhang *et al*.^28^	MoS_2_	Spatially graded *p-n* junction with bias dependent barrier height using ionic liquid
Chakraborty *et al*.^27^	Bilayer Graphene	Spatially graded *p-n* junction with bias dependent barrier height using solid polymer electrolyte
He *et al*.^26^	Graphene (CVD)	*p-n-p* junction using ionic liquid and electrostatic back gate. Junction is abrupt but each region is a few millimetres wide and spatial control of the geometry is not possible.
Our Work	Bilayer graphene	Hybrid gating (combination of electrostatic and electrolytic gating) using ionic liquid, formation of abrupt junction and photoresponse study.

To demonstrate the *p-n* junction we measure the gating curve and observe two Dirac peaks. We also study the electrical properties of the *p-n* junction and the photoresponse as a function of the junction barrier height and temperature. We find that the photoresponse is dominated by the photothermoelectric effect, characterized by a sixfold pattern in the photovoltage, similar to the results obtained with electrostatic dual gates^29^. The photovoltage increases as the temperature decreases which is indicative of hot electron thermalization by disorder assisted supercollisions^30^.

## Experiment

Graphene flakes identified with visual contrast and Raman spectroscopy (Supplementary Section I) were mechanically exfoliated on Si/SiO_2_ (300 nm) chips and metallization with titanium (7 nm) and gold (85 nm) is done using standard electron beam lithography. The ionic liquid reacts with electrodes made from chromium and gold and this limits the metals that can be used. A large in-plane electrode is made in the vicinity of graphene to serve as the top gate electrode.

In order to mask the graphene to protect it from the ionic liquid, we have tried partial coverage with polymethylmethacrelate (PMMA) resist, with overexposed PMMA, and HSQ resist. We found that PMMA tended to dissolve in the ionic liquid over time and LiClO_4_ in a PEO matrix was not sufficiently optically transparent (Supplementary Section II). Multiple electrodes are present on each of the regions so that the the electrical properties can be separately measured and we can verify that the resist protects graphene from ionic gating. We have used a thick (500 nm) HSQ protective layer patterned with a dose of 350 *μ*C/cm^2^. We have also fabricated devices completely covered with HSQ in order to verify that they are not affected by the ionic top gate (Supplementary Section III).

**Figure 2:**
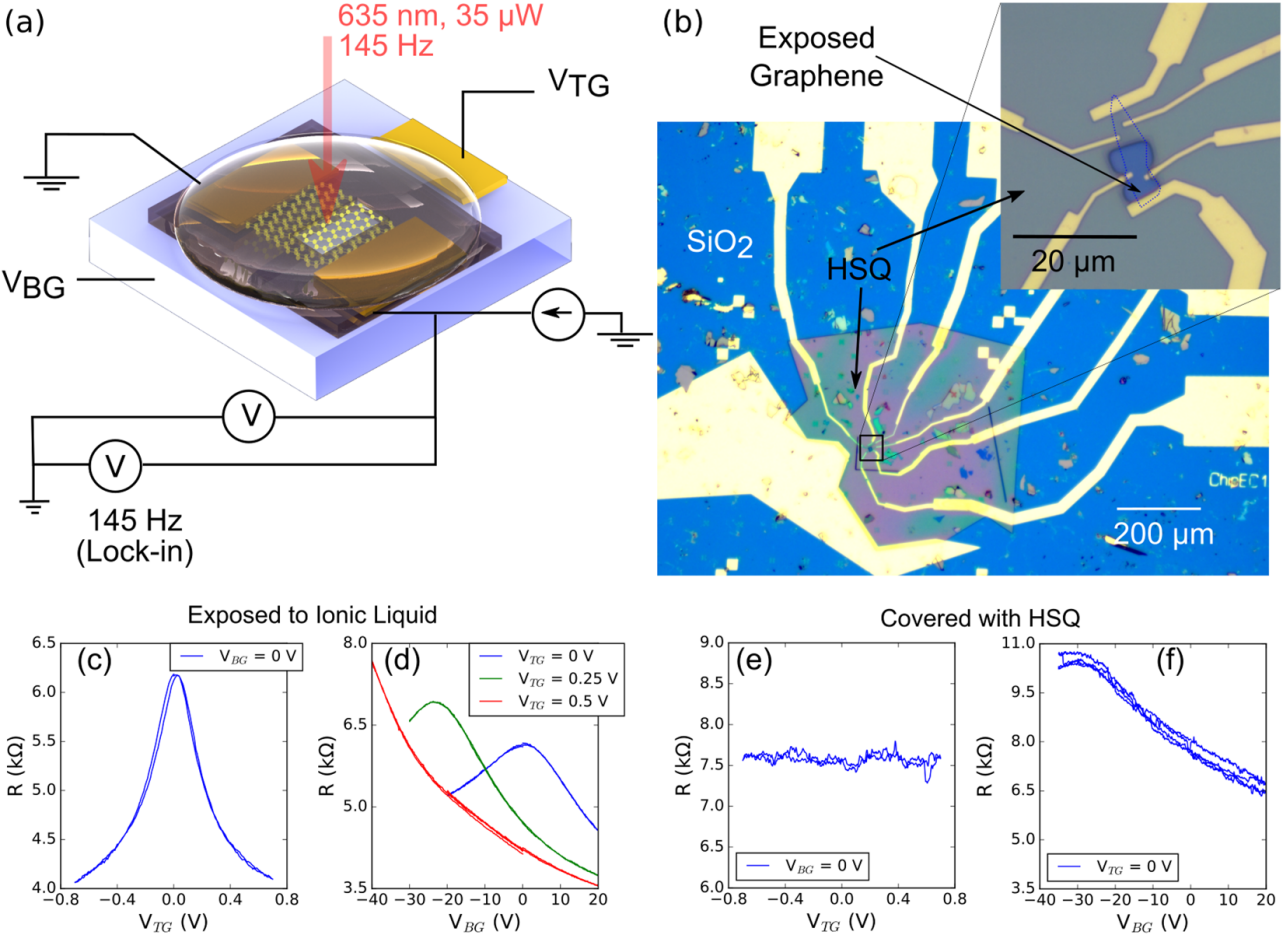
Optical image and resistance measurements. **(a)** Schematic of a graphene device which is gated both electrostatically and electochemcically and the measurement circuit with simultaneous measurement of resistance and photovoltage. (Supplementary Section VIII). **(b)** Optical image of the graphene device on which measurements have been performed. The substrate is silicon with 300 nm of silicon dioxide. Source and drain electrodes, and a top gate electrode which is large in comparison with graphene, are made from titanium and gold. The graphene flake is partially masked with 500 nm thick HSQ. The ionic liquid drop is small and does not touch the aluminium wire bonds. **(c**,**d**,**e**,**f)** Resistance measurements as a function of top gate and back gate voltages at 280 K measured separately for the exposed and covered regions. **(c)** and **(d)** Resistance variation with the top gate and back gate in the region exposed to the ionic liquid. This region is influenced by both the gates. **(e)** and **(f)** Resistance variation with the top and back gates in the region covered with HSQ. The absence of gating with the top gate shows that HSQ is able to shield part of the graphene flake.

An optical image of the device used and the measurement scheme is shown in Fig. 2. Measurements are performed in an optical cryostat with simultaneous measurement of the resistance and photovoltage.

Ionic liquids^31^ are salts that are liquid at room temperature, are transparent and stable. We have used the ionic liquid EMI-Im (also called EMI-TFSI). Its glass transition temperature^32^ is 175 K and melting point^33^ is 258 K. Below the freezing point, the ions in ionic liquid are immobilized and do not respond to externally applied fields. Our measurements are conducted above (273 K) and below (30–150 K) the freezing point. Changing the voltage applied to the ionic gate at *T* ≤ 150 K is done by cycling the temperature. Ionic liquids are hygroscopic^34^ and their electrical properties are degraded by water absorption. Dehydration is usually done by heating in vacuum or by freeze drying^35^. Our measurements are performed in an optical cryostat, and the device is initially cooled to around 30 K in vacuum, leading to removal of water.

We have noticed that it is necessary to put a very small amount of ionic liquid so that the size of the droplet is small. When the ionic liquid drop covers a large area, the back gate capacitance increases, similar to the effect seen by Xia *et al*.^36^. We have estimated that the effective increase in back gate capacitance is by factor of ~7 for a drop size of ~1 mm^2^ (Supplementary Section IV). Besides this, a large ionic liquid drop can touch the aluminium wire bonds with which it reacts. We use a 25 *μm* wire-bonder wire to wick a small amount of the ionic liquid and put in on the graphene device. The HSQ surface, in contrast with overexposed PMMA tends to repel the ionic liquid and this can be an added factor in the ionic liquid preferentially covering only the exposed parts of graphene. The refractive index of the ionic liquid and HSQ are nearly identical (~1.5) so that a surface of uniform refractive index is presented to the incident light (Supplementary Figure S5).

We have also monitored the current through the top gate using a sourcemeter to find the usable electrical limits of the top gate voltage (Supplementary Section V). A top gate voltage of ±2.5 V results in a current less than 1 nA. This current does not change if we turn on the laser illumination, indicating an absence of photochemical reactions. After applying a top gate voltage of 4 V, we have observed that the Ti/Au electrodes directly under the ionic liquid were corroded and the ones protected by HSQ were intact, further indicating that HSQ is able to effectively shield the underlying material from the ionic liquid (Supplementary Figure S5).

Figure 2 shows resistance measurements at 280 K as a function of the two gates for each of the exposed and masked region separately. The region which is exposed to the ionic liquid shows gating with both the top and back gates. We have calculated the ionic gate capacitance using the back gate capacitance of 11.5 *nF/*cm^2^ and the capacitance ratio *η* = *C*_*TG*_/*C*_*BG*_ ~ 90; this gives the ionic gate capacitance *C*_*TG*_ = 1 *μF/*cm^2^. We observe that the resistance of the region of the flake which is masked by HSQ remains unaffected by the top gate. All subsequent measurements are done across the *p-n* junction.

**Figure 3:**
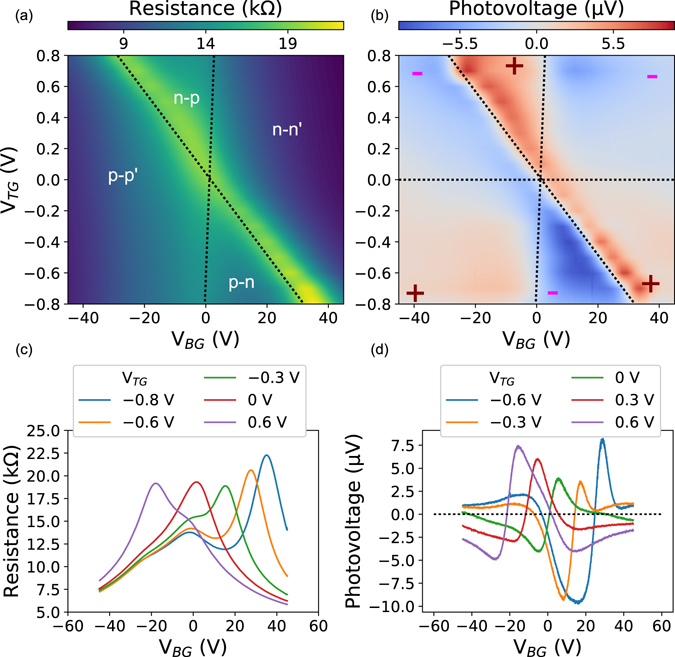
Photovoltage and resistance measurements at 120 K. To change the top gate voltage, the device is warmed to 273 K and the top gate voltage is adjusted and allowed to stabilize for half an hour before the device is cooled down to 120 K. **(a)** The measured resistance as a function of both gates. The dotted lines indicate the charge neutrality peaks of the two regions and the polarity of the exposed and covered regions is indicated. **(b)** The corresponding photovoltage at the junction measured simultaneously with the resistance. There is a clear signature of photothermoelectric effect indicated by the sixfold pattern^29^. Six regions with alternating positive and negative signs are indicated by plus and minus signs and dotted lines that separate the six regions have been drawn. **(c)** Slices of the resistance data in Fig. 3(a) exhibit two Dirac peaks. **(d)** Slices of the photovoltage with multiple zero crossings.

The variation of the resistance across the *p-n* junction at 273 K for all values of |*V*_*TG*_| ≤ 0.8 V and *V*_*BG*_ ≤ 25 V is shown in Supplementary Section VI and indicates the formation of *p-n* junction at room temperature. Similar electrical measurements performed at 120 K are shown in Fig. 3. These consist of four regions corresponding to *p-n*, *n-p*, *n-n* and *p-p* doping. The dotted lines indicate the charge neutrality peaks of the two regions and the polarity of the exposed and covered regions is indicated. The maximum carrier density was determined by using the known value of the ionic gate capacitance and the maximum gate voltage applied and it is found to be 7 × 10^12^ cm^−2^.

To change the top gate voltage, the device was first warmed to 273 K and the top gate voltage is adjusted and allowed to stabilize for 30 minutes before the device is cooled down to 120 K. The photovoltage generated at 120 K at the *p-n* junction is given in Fig. 3 along with line slices of the data for given values of the top gate voltage. The data has been acquired by repeated warming and cooling for each value of the top gate voltage. The junction is robust and does not show any thermal cycling effects (Supplementary Section IX).

The plot of the photovoltage at the junction shown in Fig. 3(b) as a function of the top and back gate voltages exhibits a six-fold pattern. The photovoltage is zero along the lines *n*_*TG*_ = 0, *n*_*BG*_ = 0 and *n*_*TG*_ = *n*_*BG*_ and alternatively positive and negative on either side (*n*_*TG*_ and *n*_*BG*_ are the carrier densities in the region exposed to the ionic liquid and covered with HSQ respectively). This manifests itself as two intersections of the photoresponse curve with zero when measuring the photovoltage as a function of the each gate, a shown in Fig. 3(c). The conventional mechanism of photoresponse generation in semiconductors is the generation of an electron hole pair due to the the absorption of a photon and their separation under the influence of a built-in electric field such as that arising at a *p-n* junction. This is referred to as the photovoltaic effect and the net photoresponse depends on the magnitude and direction of band bending.

**Figure 4:**
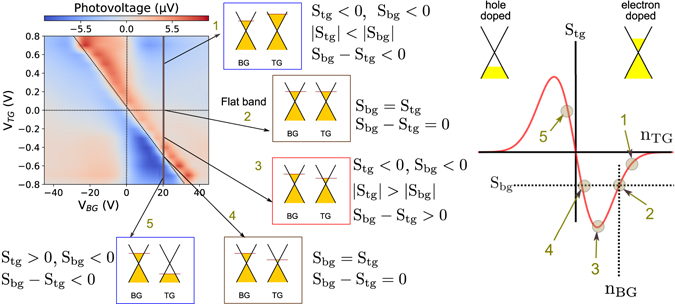
The magnitude and signs of the Seebeck coefficient that leads to the six-fold pattern seen in the photovoltage data. Along the constant *V*_*BG*_ line, the charge density in the covered region is constant and the exposed regions changes. The photovoltage is zero at point 2 and 4 where Seebeck coefficients are equal. The photovoltaic response is zero at point 2, which is the flat band condition where the Fermi levels in both regions are equal. At point 4, the photoresponse is zero despite unequal Fermi levels and provides evidence of the photothermoelectric effect. The plot on the right schematically shows the variation of *S*_*tg*_ with *n*_*tg*_ at fixed values of *S*_*bg*_ and *n*_*bg*_. The existence of two distinct values of *n*_*tg*_ where *S*_*tg*_ = *S*_*bg*_ leads to the two photoresponse zeros seen in Fig. 3(d).

We schematically illustrate in Fig. 4 that the photovoltaic effect cannot explain the multiple polarity reversals that are exhibited by the *p-n* junction. Along the highlighted line shown in Fig. 4 with a constant value of *V*_*BG*_, the charge density in the region covered with HSQ remains constant. Along this line, only the Fermi level of the exposed region changes. This change is monotonic and the photoresponse will only change sign once by going through zero at the flat band condition at point 2. However, the experimentally measured photoresponse data goes through zero twice, at point 2 and point 4, and therefore cannot be explained by the photovoltaic effect.

The photothermoelectric effect is consistent with our observations of a six-fold pattern. Under the effect of light illumination, the temperature at the *p-n* junction increases and the temperature at the source and drain contacts remains at the bath temperature. The difference in carrier densities on either side of the junction leads to different Seebeck coefficients in the two regions. This results in a net photo-induced voltage developing across the device.

The multiple polarity reversals arise because of the functional form of the Seebeck coefficient’s dependence on the charge density, illustrated in Fig. 4. The Seebeck coefficient is positive for hole doping and negative for electron doping. It is an odd function and goes through a maximum before decreasing at large carrier densities. For a given value of the Seebeck coefficient of the covered region influenced by the back gate, there are two distinct carrier densities where Seebeck coefficient of the exposed top-gated region will be equal to that of back gated region, resulting in a zero photovoltage. Along the highlighted line in Fig. 4, the sign and magnitude of the Seebeck coefficient and the photovoltage at five distinct points is highlighted.

The photovoltage is given by difference in the Seebeck coefficients in the two regions:

1 $$PV({V}_{BG},{V}_{TG})=[S({n}_{BG})-S({n}_{TG})]{\rm{\Delta }}T$$

and the Mott relation relates the Seebeck coefficient and the charge density:

2 $$S=\frac{{\pi }^{2}{k}_{B}^{2}T}{3e}\frac{d(lnR)}{dn}\frac{dn}{dE}$$

For bilayer graphene^27^, $n=-{sign}({E}_{F})\alpha [{\gamma }_{1}|{E}_{F}|+{E}_{F}^{2}]$ and $dn/dE=-\sqrt{{\alpha }^{2}{\gamma }^{2}+\mathrm{4|}n|\alpha }$, where *n* < 0(*n* > 0) for electron (hole) doping, $\alpha ={(\pi {(\hslash {v}_{f})}^{2})}^{-1}$, and the inter-layer hopping energy *γ*_1_~390 meV. The resistance data is fitted to the form $R=L/W{(e\mu )}^{-1}\frac{1}{\sqrt{{n}^{2}+{n}_{0}^{2}}}$, from which we get $d(lnR)/dn=\frac{-n}{{n}^{2}+{n}_{0}^{2}}$.

**Figure 5:**
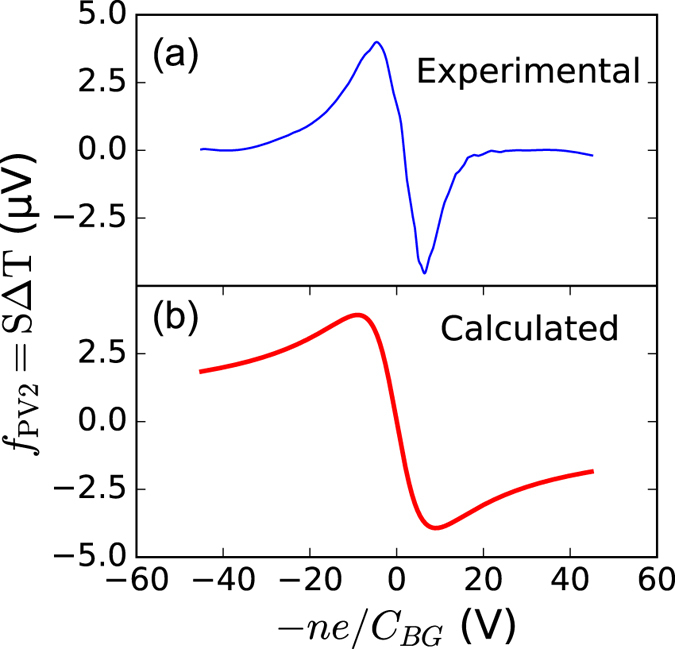
A comparison of the product of the Seebeck coefficient and temperature increase obtained experimentally and calculated theoretically as a function of the carrier density. **(a)** The experimental curve has been obtained by Fourier transforming the data in Fig. 3(a) and represents the function *f*_*PV*2_, which corresponds to the region exposed to the top gate. **(b)** The theoretical curve has been calculated by calculating the Seebeck coefficient using the Mott relation and with a temperature increase of 50 mK.

We can estimate the value of the Seebeck coefficient from the experimental data. The photovoltage arising form the photothermoelectric can be written as a linear combination of two functions that depend on the doping level in each region, similar to equation 1:

3 $$PV={f}_{PV1}({V}_{BG})-{f}_{PV2}({V}_{BG}+\eta ({V}_{TG}))$$

where *η* = *C*_*TG*_/*C*_*BG*_ and we treat the functions *f*_*PVi*_ as unknowns and estimate them from the experimental data using two dimensional Fourier transforms (Supplementary Section VII), similar to the approach taken by Gabor *et al*.^29^. The function *f*_*PV*2_ has been plotted in Fig. 5(a) as a function of the carrier density. For the photothermoelectric effect, the function is related to the Seebeck coefficient as *f*_*PVi*_ = *S*Δ*T* (S is the Seebeck coefficient and Δ*T* the temperature increase). The experimental value is compared to the theoretical result obtained using the Mott relation, equation 2 and is shown in Fig. 5(b).

The temperature increase is estimated from the experimental data as Δ*T* = *f*_*PV*_/*S* = 50 mK. This is smaller than the expected temperature increase of ~1 *K* that would be expected from the electronic thermal conductivity of graphene^37^ of ~2–5 W/m/K, also obtainable from the the Wiedemann-Franz relation. This difference is because the temperature increase of the electronic subsystems obtained through the heat balance equation^30^ is strongly dependent on the cooling length. We have also neglected any light absorption in the ionic liquid and the effective optical power reaching the device and the corresponding temperature increase could be lower.

**Figure 6:**
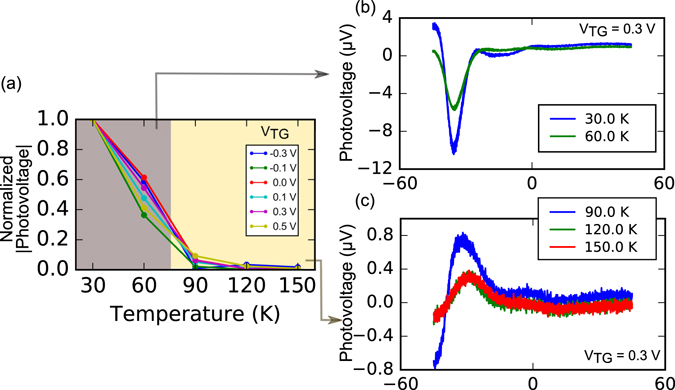
Temperature dependence of the photovoltage. **(a)** Normalized photovoltage magnitude as a function of temperature at different top gate voltages when *n*_*BG*_ = −*n*_*TG*_. The normalization factor depends on *V*_*TG*_. **(b)** and **(c)** The photovoltage as a function of the back gate at *V*_*TG*_ = 0.3 V.

Photovoltage measurements at temperatures ranging from 30 K to 150 K have also been performed. The normalized photovoltage magnitude at different top gate voltages has been plotted in Fig. 6(a). The photovoltage increases with decreasing temperature. The photovoltage rises rapidly below 90 K and in this range, the product of photovoltage and temperature is constant, indicating that the photovoltage is inversely proportional to temperature (Supplementary Section X). The photo-responsivities we have obtained range from 100 mV/W at 273 K to 600 mV/W at 30 K. Increase in the photovoltage at lower temperatures indicates an increase in the cooling length, implying that the cooling rate increases with temperature. This is similar to the trend observed^38^ in monolayer graphene where this has been attributed to disorder mediated supercollisions^30, 39, 40^ as the dominant electronic thermalization mechanism.

In conclusion, we have achieved the formation of an abrupt *p-n* junction in graphene using a combination of electrostatic and electrolytic gating using HSQ as a protective mask. This technique is scalable and the fabrication of an array of *p-n* junctions, such as a superlattice^41^, can be realized by the narrow features that can be lithographically patterned using HSQ. The combination of larger optical transparency of ionic liquids and the potential for larger carrier densities make this an interesting system for optoelectronic studies.

## Electronic supplementary material

Supplementary Information

## References

[1] Novoselov KS (2004). Electric field effect in atomically thin carbon films. Science.

[2] Koppens FHL (2014). Photodetectors based on graphene, other two-dimensional materials and hybrid systems. Nat Nano.

[3] Bonaccorso F, Sun Z, Hasan T, Ferrari AC (2010). Graphene photonics and optoelectronics. Nature Photonics.

[4] Bao Q, Loh KP (2012). Graphene photonics, plasmonics, and broadband optoelectronic devices. ACS Nano.

[5] Castro Neto AH, Peres NMR, Novoselov KS, Geim AK (2009). The electronic properties of graphene. Reviews of Modern Physics.

[6] Das A (2008). Monitoring dopants by raman scattering in an electrochemically top-gated graphene transistor. Nature nanotechnology.

[7] Petach, T. a., Lee, M., Davis, R. C., Mehta, A. & Goldhaber-Gordon, D. Mechanism for the large conductance modulation in electrolyte-gated thin gold films. *Physical Review B* **90**, 081108(R), doi:10.1103/PhysRevB.90.081108 (2014).

[8] Shimotani H, Asanuma H, Takeya J, Iwasa Y (2006). Electrolyte-gated charge accumulation in organic single crystals. Applied Physics Letters.

[9] Vanmaekelbergh D, Houtepen AJ, Kelly JJ (2007). Electrochemical gating: A method to tune and monitor the (opto)electronic properties of functional materials. Electrochimica Acta.

[10] Rosenblatt S (2002). High performance electrolyte gated carbon nanotube transistors. Nano Letters.

[11] Ueno K (2008). Electric-field-induced superconductivity in an insulator. Nature Materials.

[12] Bollinger AT (2011). Superconductor–insulator transition in La2-xSrxCuo4 at the pair quantum resistance. Nature.

[13] Farmer, D. B., Lin, Y. M., Afzali-Ardakani, A. & Avouris, P. Behavior of a chemically doped graphene junction. *Applied Physics Letters* **94** (2009).

[14] Peters EC, Lee EJH, Burghard M, Kern K (2010). Gate dependent photocurrents at a graphene p-n junction. Applied Physics Letters.

[15] Yu X, Shen Y, Liu T, Wu TT, Jie Wang Q (2015). Photocurrent generation in lateral graphene p-n junction created by electron-beam irradiation. Scientific reports.

[16] Seo BH, Youn J, Shim M (2014). Direct laser writing of air-stable p–n junctions in graphene. ACS Nano.

[17] Kim YD (2013). Focused-laser-enabled p–n junctions in graphene field-effect transistors. ACS Nano.

[18] Lin L (2016). Tuning chemical potential difference across alternately doped graphene p–n junctions for high-efficiency photodetection. Nano Letters.

[19] Yan K (2012). Modulation-doped growth of mosaic graphene with single-crystalline p-n junctions for efficient photocurrent generation. Nature communications.

[20] Wang S, Sekine Y, Suzuki S, Maeda F, Hibino H (2015). Photocurrent generation of a single-gate graphene p–n junction fabricated by interfacial modification. Nanotechnology.

[21] Huang Y (2015). Reliable exfoliation of large-area high-quality flakes of graphene and other two-dimensional materials. ACS Nano.

[22] Chen F, Qing Q, Xia J, Li J, Tao N (2009). Electrochemical gate-controlled charge transport in graphene in ionic liquid and aqueous solution. Journal of the American Chemical Society.

[23] Kim BJ (2010). High-performance flexible graphene field effect transistors with ion gel gate dielectrics. Nano Letters.

[24] Ye JT (2012). Superconducting dome in a gate-tuned band insulator. Science.

[25] Efetov DK, Kim P (2010). Controlling electron-phonon interactions in graphene at ultrahigh carrier densities. Physical Review Letters.

[26] He, X. *et al*. Formation of p-n-p junction with ionic liquid gate in graphene. *Applied Physics Letters* **104** (2014).

[27] Chakraborty B, Das A, Sood AK (2009). The formation of a p-n junction in a polymer electrolyte top-gated bilayer graphene transistor. Nanotechnology.

[28] Zhang YJ, Ye JT, Yomogida Y, Takenobu T, Iwasa Y (2013). Formation of a stable p–n junction in a liquid-gated MoS_2_ ambipolar transistor. Nano Letters.

[29] Gabor NM (2011). Hot carrier-assisted intrinsic photoresponse in graphene. Science.

[30] Song JCW, Reizer MY, Levitov LS (2012). Disorder-assisted electron-phonon scattering and cooling pathways in graphene. Physical Review Letters.

[31] Galiński M, Lewandowski A, Stȩpniak I (2006). Ionic liquids as electrolytes. Electrochimica Acta.

[32] McEwen AB (1999). Electrochemical properties of imidazolium salt electrolytes for electrochemical capacitor applications. Journal of The Electrochemical Society.

[33] Ngo HL, LeCompte K, Hargens L, McEwen AB (2000). Thermal properties of imidazolium ionic liquids. Thermochimica Acta.

[34] Welton T (1999). Room-temperature ionic liquids. solvents for synthesis and catalysis. Chemical Reviews.

[35] Wellens S, Thijs B, Binnemans K (2013). How safe are protic ionic liquids? explosion of pyrrolidinium nitrate. Green Chemistry.

[36] Xia JL, Chen F, Wiktor P, Ferry DK, Tao NJ (2010). Effect of top dielectric medium on gate capacitance of graphene field effect transistors: Implications in mobility measurements and sensor applications. Nano Letters.

[37] Yiǧen S, Champagne AR (2014). Wiedemann–franz relation and thermal-transistor effect in suspended graphene. Nano Letters.

[38] Ma Q (2014). Competing channels for hot-electron cooling in graphene. Physical Review Letters.

[39] Graham MW, Shi S-F, Ralph DC, Park J, McEuen PL (2013). Photocurrent measurements of supercollision cooling in graphene. Nature Physics.

[40] Betz AC (2012). Hot electron cooling by acoustic phonons in graphene. Physical Review Letters.

[41] Dubey S (2013). Tunable Superlattice in Graphene To Control the Number of Dirac Points. Nano Letters.

