# Supplementary material for: Abrupt p-n junction using ionic gating at zero-bias in bilayer graphene
Source: Sci Rep. 2017 Jun 13;7:3336. doi: 10.1038/s41598-017-03264-0 (PMC5469745; doi:10.1038/s41598-017-03264-0)
Supplement: Supplementary file 1 — Supplementary Information [file 41598_2017_3264_MOESM1_ESM.pdf]

Abrupt *p*-*n* junction using ionic gating at zero-bias in bilayer graphene

Sameer Grover,[1] Anupama Joshi,[1,2] Ashwin Tulapurkar,[2] and Mandar M. Deshmukh[1,*]

[1]*Department of Condensed Matter Physics and Materials Science,*
Tata Institute of Fundamental Research,
Homi Bhabha Road, Mumbai 400005, India

[2]*Department of Electrical Engineering,*
Indian Institute of Technology Bombay, Mumbai 400076, India

(Dated: March 18, 2017)

* deshmukh@tifr.res.in

I. RAMAN SPECTRUM OF GRAPHENE

Figure S1 shows the Raman spectrum of the bilayer graphene flake presented in the main text measured with an excitation wavelength of 532 nm.

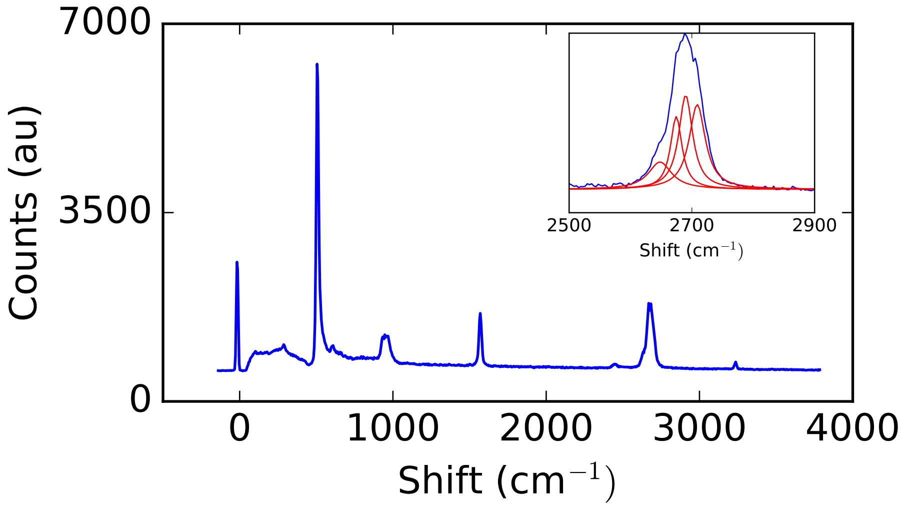

FIG. S1. Raman spectrum of bilayer graphene flake shown in Figure 1 in the main text

II. OPTICAL TRANSPARENCY OF $LICLO_4$/PEO

We tried using a PEO/$LiClO_4$ solid electrolyte but found that it was not optically transparent. Though it has been used before for optical measurements [1], it is difficult to apply it locally so that it is uniform and does not contact the back gate.

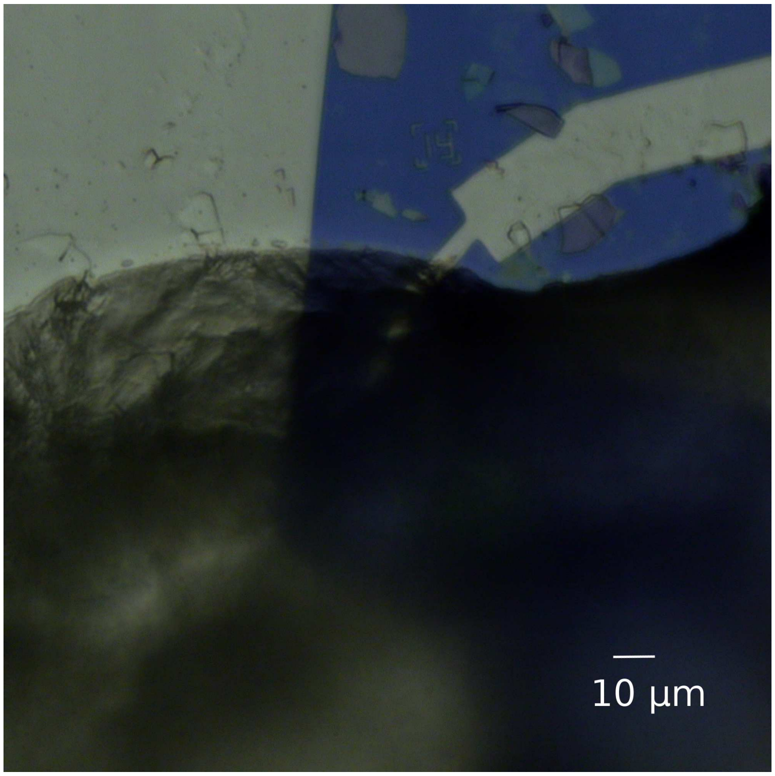

FIG. S2. Optical image of a graphene device covered with a solid electrolyte made from $LiClO_4$/PEO.

III. IONIC GATE SCREENING BY HSQ AND PMMA

In order to verify whether HSQ is able to effectively mask the ionic liquid, we fabricated a graphene device which was completely covered with HSQ. The measured resistance as a function of both the top gate and back gate separately is given in Figure S3.

Even though the device is fully covered with HSQ, Figure S3(b) shows that the change in the top gate voltage causes the resistance to change slightly. This change is small and indicative of a low capacitance. On plotting the gating curves of the top gate and back gate on the same scale (Figure S3(c)), it can be seen that the change in the resistance because of the top gate is comparable to that due to the back gate. This indicates that the capacitance between the electrolytic top gate and graphene is smaller than what would be expected from a few nanometres thick Debye layer and that the high capacitance due to the electrolyte has been suppressed.

We also made another device using PMMA overexposed at a dose of 10000 $\mu C/cm^2$ which completely covers the graphene flake. The gating curves for this device are shown in Figure S4. The resistance changes with the top gate and the slope dG/dV_{TG} is more than that of the back gate. However, the magnitude of the change is small compared to that of the back gate. It is possible that there are small areas within PMMA that the ionic liquid can percolate through and affect graphene.

We also applied large top gate voltages ($\sim$ 4.5 V) to the bilayer graphene device that is presented in the main text. This caused corrosion of the electrodes. From the optical image given in Figure S5, we find that the electrodes covered by the HSQ are unaffected, once again indicating that the HSQ is an effective mask for the ionic liquid.

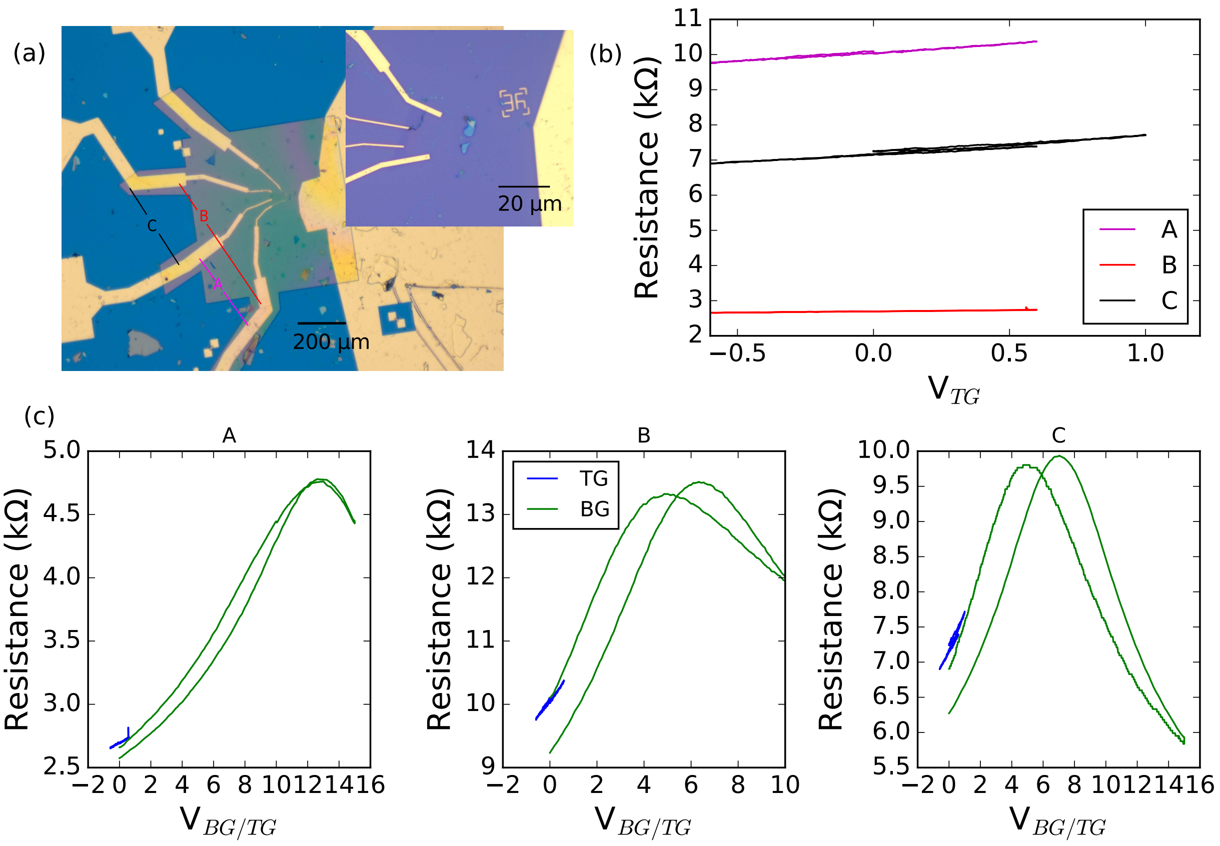

FIG. S3. (a) Optical image showing the graphene device completely covered with HSQ. (Inset) Optical image after metallization and before HSQ was deposited. Three pairs of electrodes on which measurements are done are labelled. (b) Variation of the device resistance as a function of the top gate voltage. (c) Gating with the top and back gate plotted on the same scale.

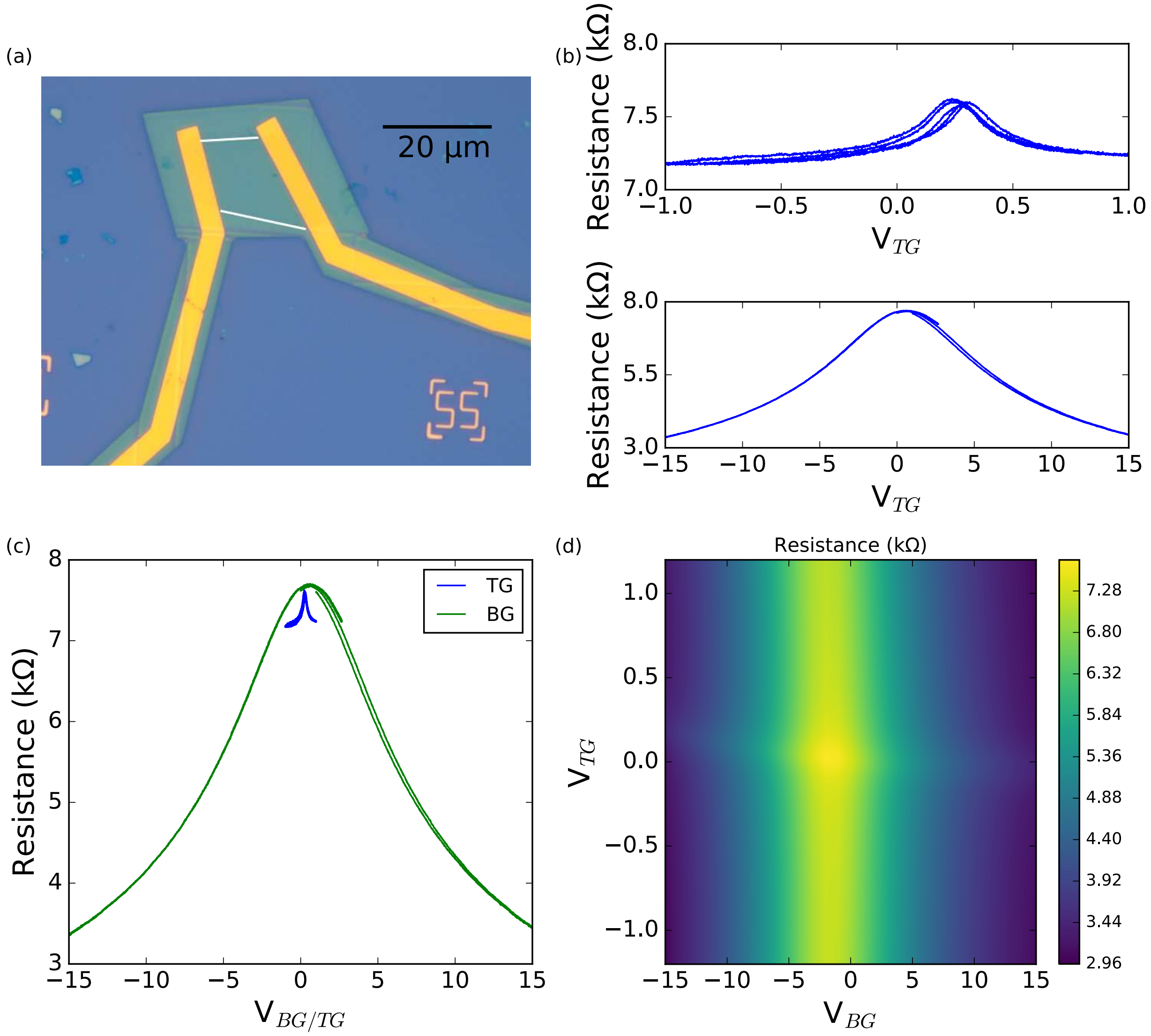

FIG. S4. (a) Optical image of the graphene device covered with PMMA. The boundaries of the flake are marked in blue. The entire region, including the electrodes are covered with overexposed PMMA. (b) The gating curves of the device with the top and back gates. (c) The two gating curves overlaid on each other. (d) The resistance as a function of top gate and back gate voltages.

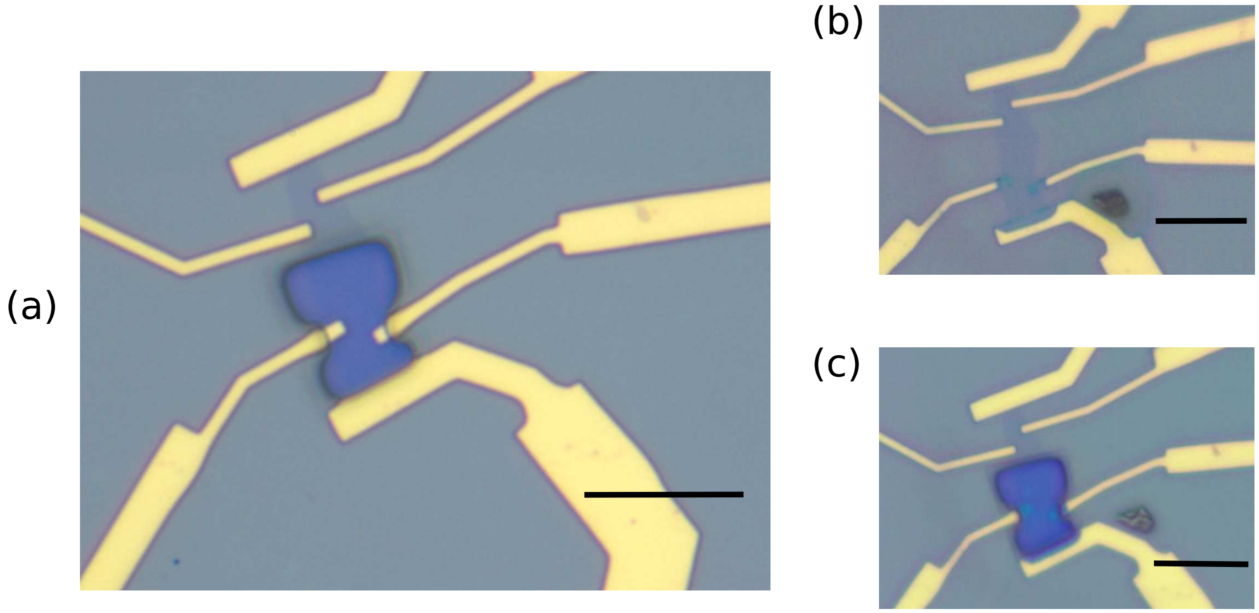

FIG. S5. Corrosion of electrodes caused by large top gate voltage (a) Optical image of device after HSQ patterning, before the ionic liquid was dropped. (b) Optical image after measurements with the ionic liquid still on the device. The boundary of HSQ is not visible because the refractive index of HSQ is identical to that of the ionic liquid. (c) Optical image after washing off the ionic liquid with isopropyl alcohol. Scale bar in all images is 10 μm.

IV. CAPACITANCE VARIATION WITH AREA OF IONIC LIQUID

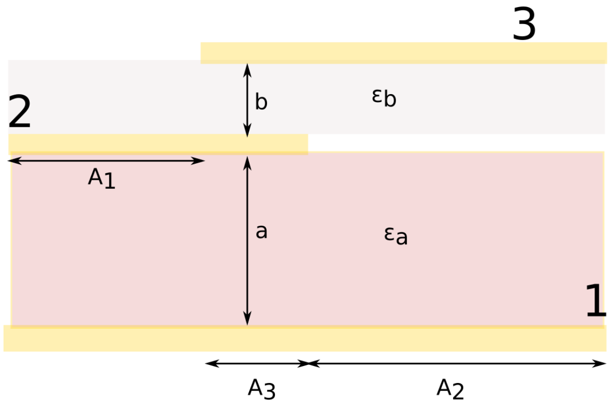

FIG. S6. Model for the dual gated FET that has been used in the calculations. The back gate, graphene and top gate are represented by the conductors 1,2 and 3 respectively. The bottom gate dielectric is SiO_2 and the electrical double layer is the top gate dielectric. The cross-sectional areas of overlap as indicated are A_1, A_2 and A_3

Ignoring the quantum capacitance by treating the graphene as a metal, we model the device as a system of conductors as shown in Figure S7. The back gate, graphene and top gate are denoted by 1, 2 and 3 and they overlap vertically with overlap areas A_i as indicated.

Neglecting fringing fields, we can write the capacitance matrix relating the charge on each conductor Q_i to its potential V_i as:

$$q_1 = \Big[\frac{A_2\epsilon_a\epsilon_b}{a\epsilon_b + b\epsilon_a} + \frac{\epsilon_a}{a}(A_1 + A_3)\Big]v_1 + \Big[-\frac{\epsilon_a}{a}(A_1 + A_3)\Big]v_2 + \Big[-\frac{A_2\epsilon_a\epsilon_b}{a\epsilon_b + b\epsilon_a}\Big]v_3 \tag{S1}$$

$$q_2 = \Big[-\frac{\epsilon_a}{a}(A_1 + A_3)\Big]v_1 + \Big[\left(\frac{A_3\epsilon_b}{b} + \frac{\epsilon_a}{a}(A_1 + A_3)\right)\Big]v_2 + \Big[-\frac{A_3\epsilon_b}{b}\Big]v_3 \tag{S2}$$

$$q_3 = \Big[-\frac{A_2\epsilon_a\epsilon_b}{a\epsilon_b + b\epsilon_a}\Big]v_1 + \Big[-\frac{A_3\epsilon_b}{b}\Big]v_2 + \Big[\frac{A_2\epsilon_a\epsilon_b}{a\epsilon_b + b\epsilon_a} + \frac{A_3\epsilon_b}{b}\Big]v_3 \tag{S3}$$

The effective capacitance per unit area between the back gate and graphene with the top gate at a fixed potential is $C_{12} = q_1/v/f$ with $v_1 = v, v_2 = v_3 = 0$ with f as the area on conductor 2 on which charge accumulates ($f \approx A_1 + A_3$).

$$C_{12} = \frac{\epsilon_a}{a}\left[\frac{1}{f\,(a\epsilon_b + b\epsilon_a)}\,(A_1 a\epsilon_b + A_1 b\epsilon_a + A_2 a\epsilon_b + A_3 a\epsilon_b + A_3 b\epsilon_a)\right] \tag{S4}$$

From the device geometry, we estimate the capacitance with $\epsilon_a = 3.9\epsilon_0$, $\epsilon_b = 16.5\epsilon_0$ [2], $A_1 = (425\,\mu\text{m})^2$, $A_3 = (80\,\mu\text{m})^2$, $b = 1$ nm, $a = 300$ nm, $f = (425\,\mu\text{m})^2$.

Figure S7 gives the factor of increase in the back gate capacitance as a function of the ionic liquid drop dimension.

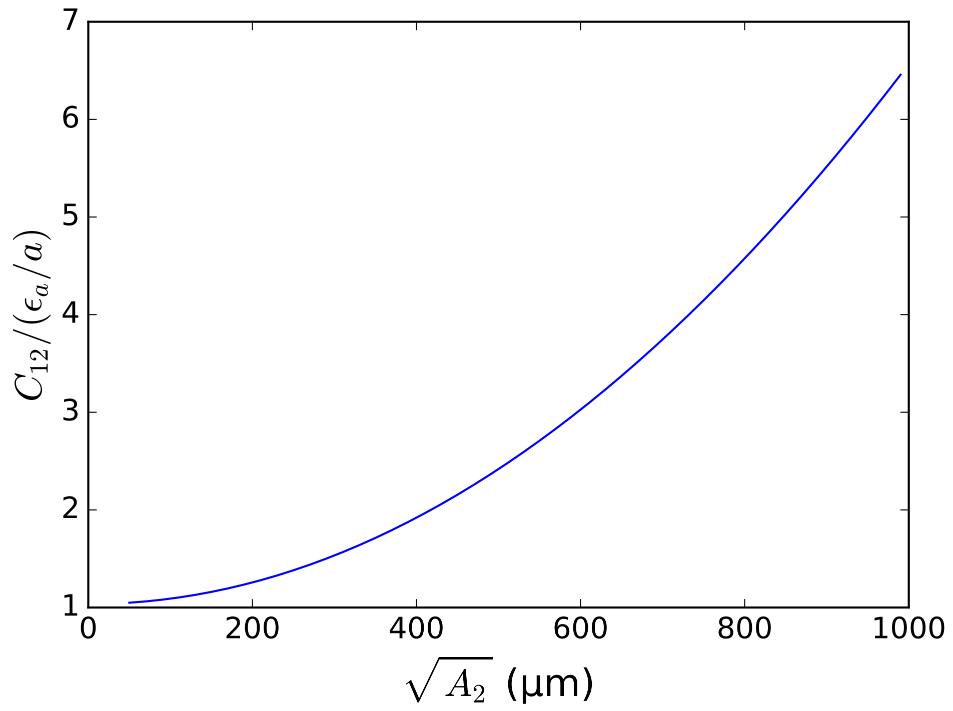

FIG. S7. Numerical solution of equation S4. The factor of the back gate capacitance increase is shown as a function of the ionic liquid drop lateral size.

V. IONIC TOP GATE LEAKAGE CURRENT

We have measured the leakage current of the ionic liquid by monitoring the current drawn by the top gate. The current is less than 1 nA till $V_{TG} = 4$ V. However, we saw an abrupt change in the device resistance at around 3.5 V

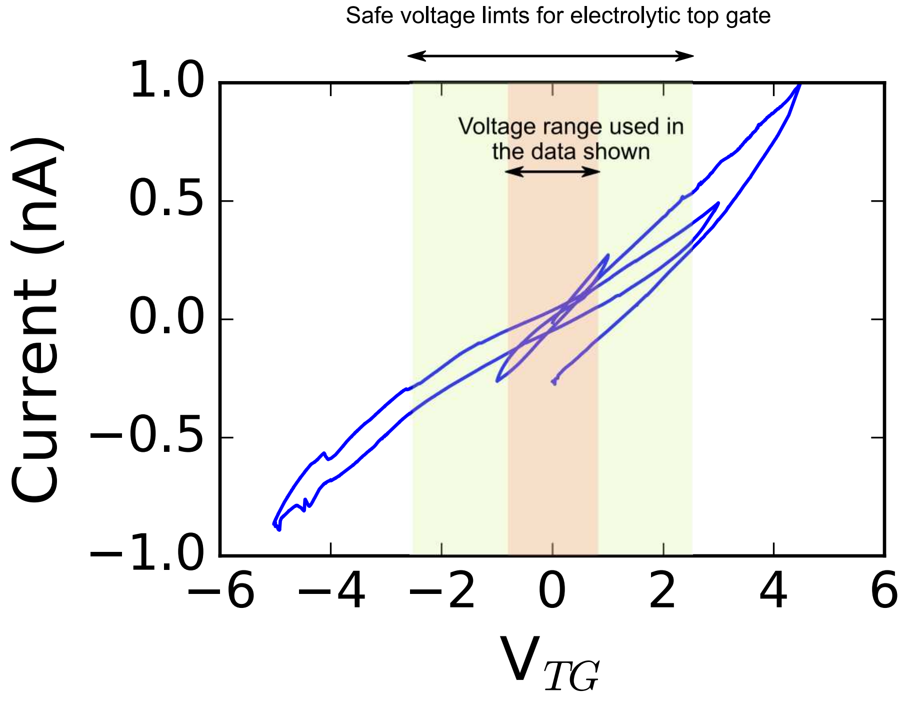

FIG. S8. The leakage current drawn by the ionic top gate as a function of the top gate voltage.

VI. ELECTRICAL CHARACTERIZATION OF *P-N* JUNCTION AT ROOM TEMPERATURE

Figure S9 shows the resistance and corresponding photovoltage at 273 K as a function of both the top and back gates.

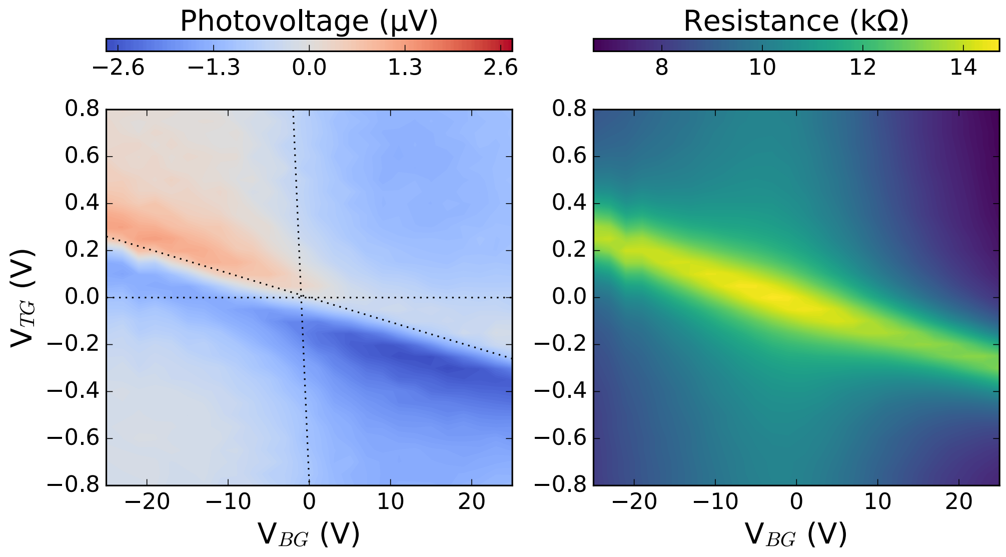

FIG. S9. Photovoltage (left) and Resistance (right) of the device at 273 K.

VII. FOURIER TRANSFORM ANALYSIS

If $z(x, y) = f(ax + b)$, $Fz(k_x, k_y) = Ff(k_x/a)\delta(ak_y - bk_x)$, where Fz and Ff are the Fourier transforms of z and f respectively and $k = 1/x$,

Accordingly, if the photovoltage PV is given by:

$$PV(V_{BG}, V_{TG}) = f_{PV1}(V_{BG}) - f_{PV2}(V_{BG} + \eta(V_{TG})) \tag{S5}$$

The Fourier transform of f_{PV1} lies along the $1/V_{BG}$ axis and f_{PV2} lies along the $1/V_{TG} = \eta(1/V_{BG})$ axis. The values of f_{PVi}, $i = 1, 2$ are found by masking with a Gaussian and taking the inverse transform.

Since, for the data at 120K, acquisition of the photovoltage at each top gate voltage involves heating and cooling, the data is sampled coarsely at intervals of 0.1 V, with 17 points ranging from -0.8 V to 0.8 V, we have only shown the function form of f_{PV2} in the main text.

VIII. MEASUREMENT CIRCUIT

Light of wavelength 635 nm modulated at 145 Hz is focused on the junction using a microscope objective. A current of 50 nA at 550 Hz is applied across the source and drain to measure the resistance. Two lock-in amplifiers, at 145 Hz and 550 Hz are used to measure the photovoltage and the resistance respectively. (see Figure 1(a) in the main text).

IX. THERMAL CYCLING STABILITY

We have verified that the p-n junction formed using the technique demonstrated in the paper is not affected by thermal cycling. We have measured both the resistance and photovoltage with before and after 15 thermal cycles between measurements taken at the same top gate voltage. Figures S15, S11 and S12 show plots of resistance taken at three different temperatures 30, 60 and 120 K for different top gate voltages. The two plots are plotted in blue and green and there are 15 thermal cycling between them. All plots show both forward and backward sweeps of the gate voltage so that hysteresis, if present, can be identified. At all three temperatures, the gating curves overlap indicating that the p-n junction created is immune to thermal cycling effects.

Similarly, the photovoltage has also been measured at 30 K and 60 K and is shown in Figures S13 and S14 , with 15 thermal cycling events between the curves. Here, the difference between the two measurement st is more pronounced. However, the trend is clearly the same. Each thermal cycling event takes a significant amount of time nearly 4 hours including the time we wait for the sample to stabilize. During this time, any position change of the sample due to thermal drift in the sample stage, or any long- term drift in the z-position of microscope objective that is used to focus light even by a few microns - will result in a change in the intensity and position of light illumination. This can account for the changes we have observe in the photovoltage cycling events shown below.

We noticed a decrease in the top gate capacitance during the first heating and cooling cycle where it decreased from $90\,C_{BG}$ to $40\,C_{BG}$ (C_{BG}: back gate capacitance) and subsequently remained stable over the course of measurements which involved more than 50 thermal cycles.

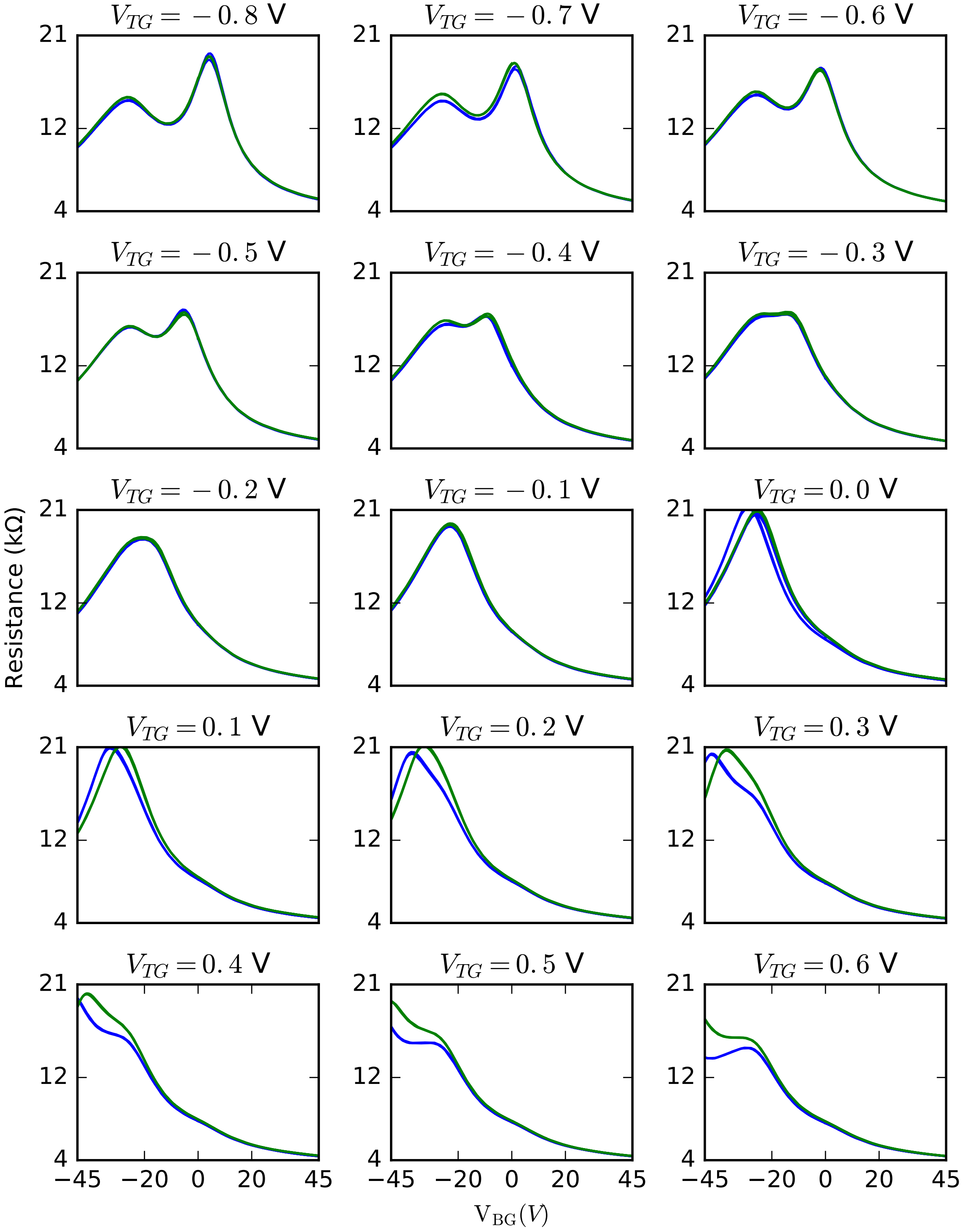

FIG. S10. Resistance at 120 K measured before and after 15 thermal cycling events.

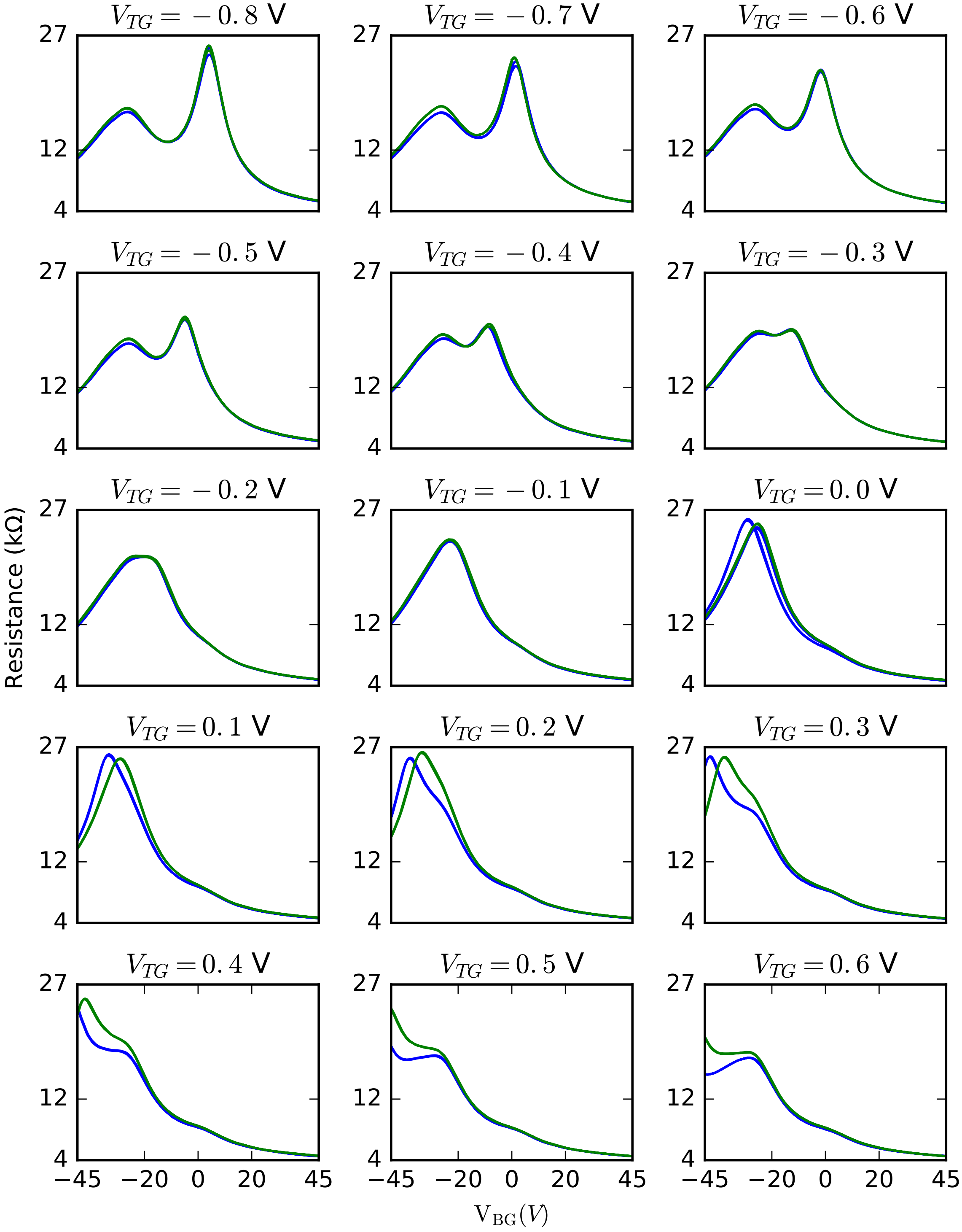

FIG. S11. Resistance at 60 K measured before and after 15 thermal cycling events.

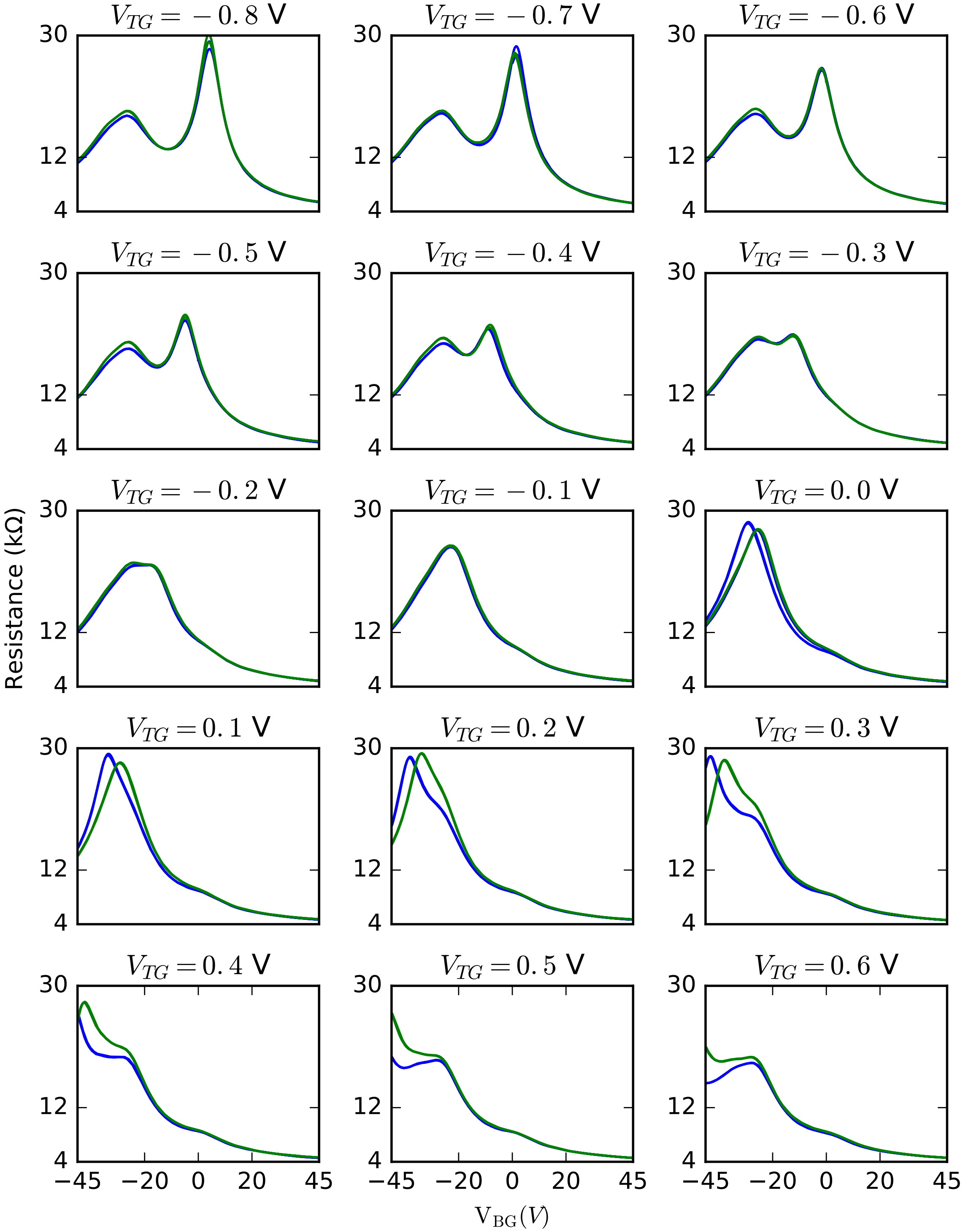

FIG. S12. Resistance at 30 K measured before and after 15 thermal cycling events.

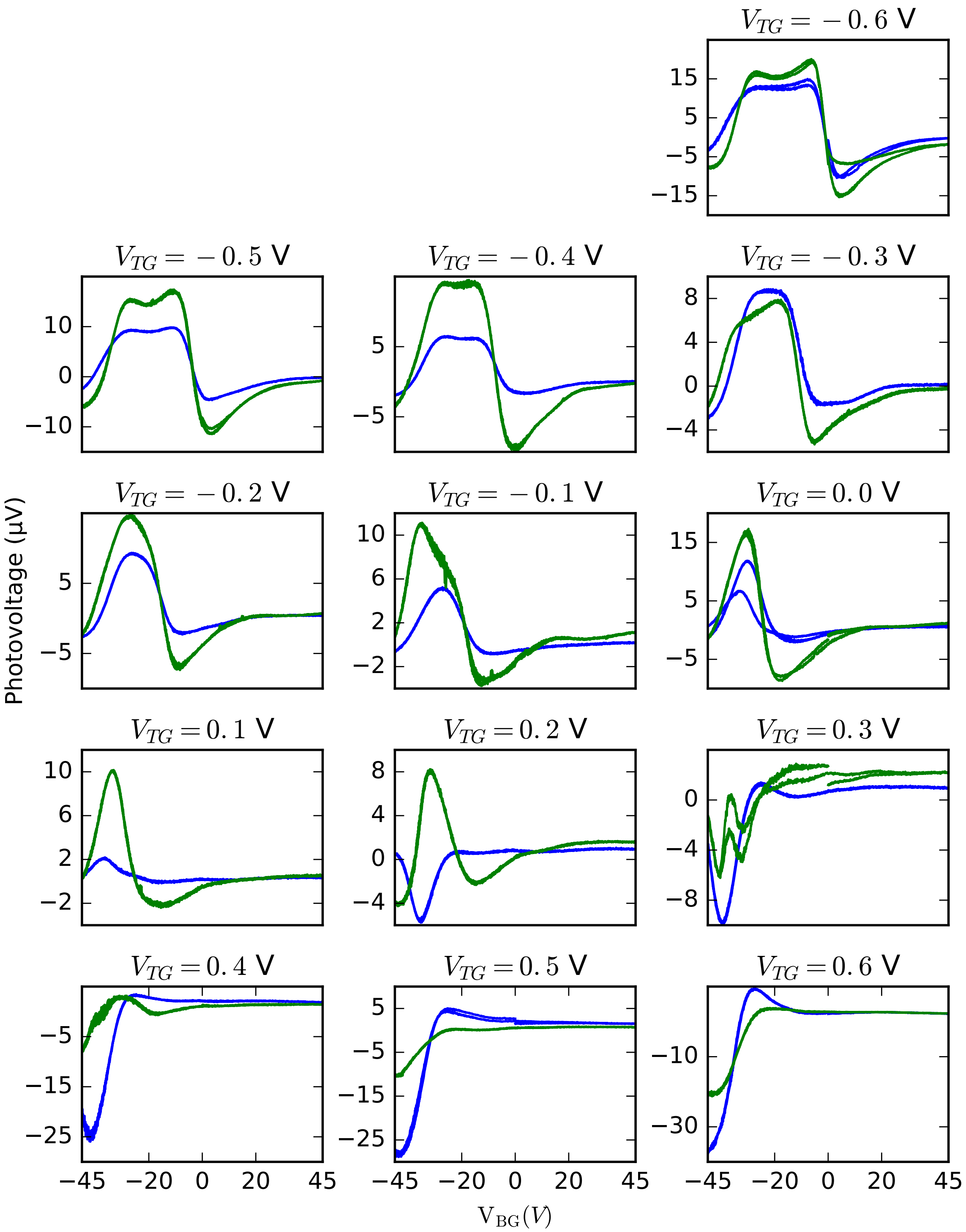

FIG. S13. Photovoltage at 60 K measured before and after 15 thermal cycling events.

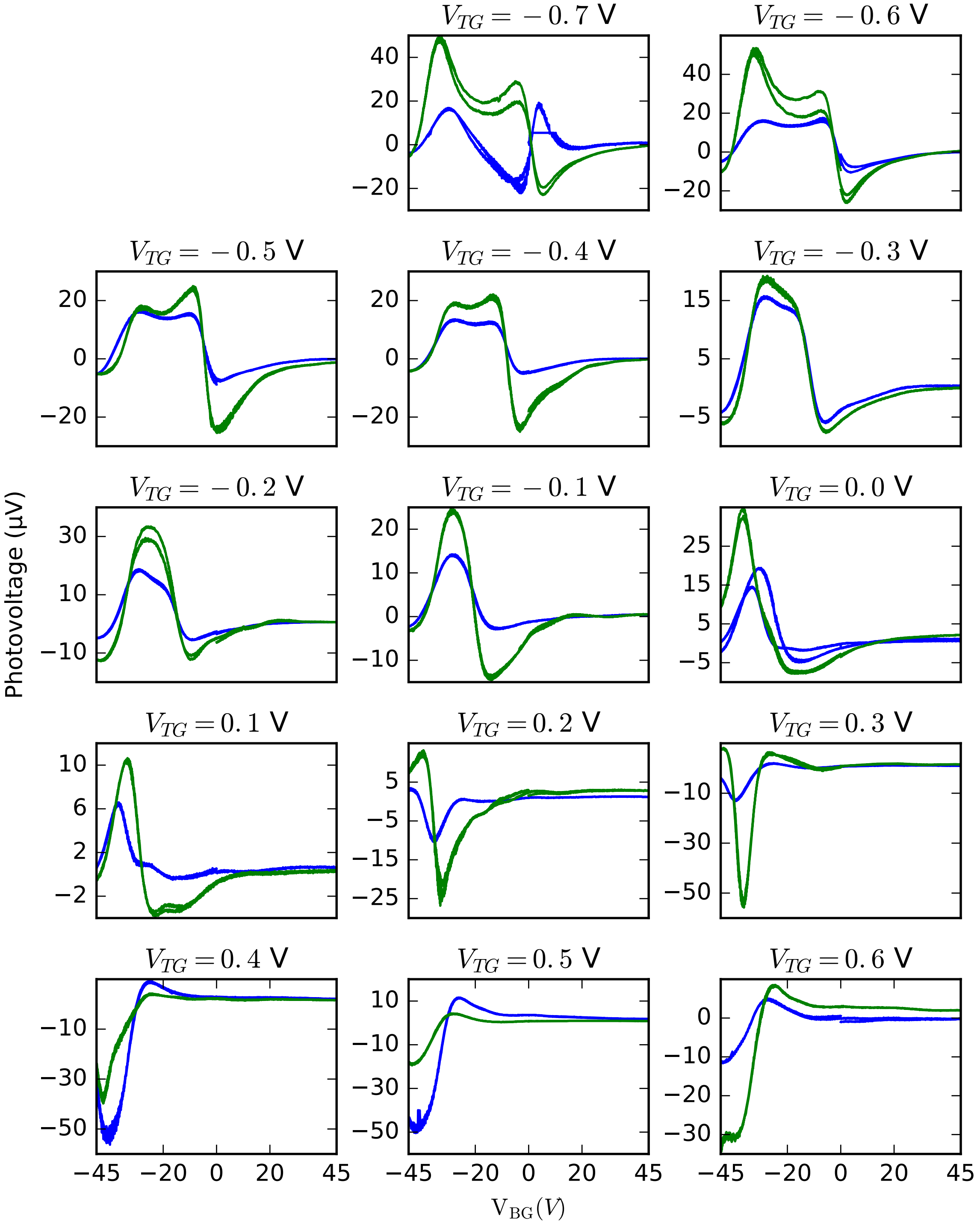

FIG. S14. Photovoltage at 30 K measured before and after 15 thermal cycling events.

X. TEMPERATURE DEPENDENCE

In Figure S15, we have plotted the product of the photovoltage and temperature as a function of the back gate voltage for different top gate voltages. Since the 30 K and 60 K curves overlap, the photovoltage $PV \propto 1/T$ in this temperature range.

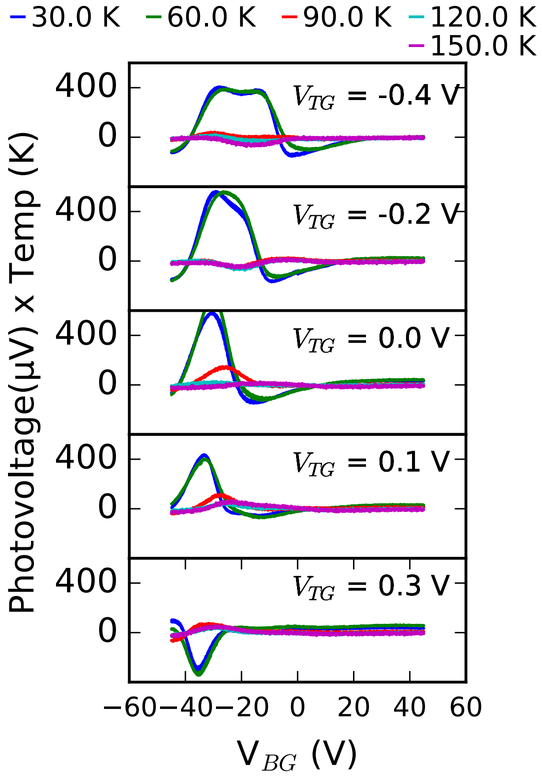

FIG. S15. Temperature dependence of the photovoltage shown by plotting the product of the photovoltage and temperature at different top gate voltages.

[1] A. Das, S. Pisana, B. Chakraborty, S. Piscanec, S. K. Saha, U. V. Waghmare, K. S. Novoselov, H. R. Krishnamurthy, a. K. Geim, A. C. Ferrari, and A. K. Sood, Nature nanotechnology **3**, 210 (2008).

[2] M.-M. Huang, Y. Jiang, P. Sasisanker, G. W. Driver, and H. Weing"artner, Journal of Chemical & Engineering Data **56**, 1494 (2011).
